# Prognostic factors of afatinib as a first-line therapy for advanced *EGFR* mutation-positive lung adenocarcinoma: a real-world, large cohort study

**DOI:** 10.18632/oncotarget.25255

**Published:** 2018-05-04

**Authors:** Sheng-Kai Liang, Meng-Rui Lee, Wei-Yu Liao, Chao-Chi Ho, Jen-Chung Ko, Jin-Yuan Shih

**Affiliations:** ^1^ Department of Internal Medicine, National Taiwan University Hospital Hsinchu Branch, Hsinchu, Taiwan; ^2^ Institute of Biotechnology, National Tsing Hua University, Hsinchu, Taiwan; ^3^ Department of Internal Medicine, National Taiwan University Hospital and College of Medicine, National Taiwan University, Taipei, Taiwan

**Keywords:** afatinib, EGFR mutation-positive lung adenocarcinoma, prognostic factor, first-line therapy, real-world study

## Abstract

Lung cancer remains the primary cause of cancer-related mortality worldwide. Several treatment modalities are available for lung cancer, including surgery, radiation, and chemotherapy. Among the chemotherapeutics available, afatinib has been shown to be effective for those with *epidermal growth factor receptor* (*EGFR*) mutation-positive lung adenocarcinoma. Herein, we analyzed the factors affecting the prognosis of patients who received afatinib as a first-line therapy for advanced *EGFR* mutation-positive lung adenocarcinoma in the real-world setting. Patients who received afatinib as a first-line therapy and were reimbursed by the National Health Insurance were recruited in this study. Data on patient characteristics and treatment courses were collected. In total, 259 patients were enrolled (median follow-up, 22.0 months). Of them, 82 (31.7%) were identified to have brain metastases at baseline, which were associated with poor Eastern Cooperative Oncology Group performance status, high incidence of central nervous system progression, and short overall survival. However, the results of our analysis showed that overall survival was not affected by reductions in the afatinib dosage or any upfront local treatments for brain tumors. Multivariate analyses showed that brain metastases at diagnosis and treatment response to afatinib are two important prognostic factors for the overall survival of patients with *EGFR* mutation-positive lung adenocarcinoma.

## INTRODUCTION

Lung cancer remains the leading cause of mortality among all malignant diseases worldwide. Despite advances in precision medicine in the past decade, the 5-year overall survival of patients with advanced lung cancer is less than 10% [1, 2]. Several favorable prognostic factors had been discussed in patients with advanced non-small cell lung cancer (NSCLC), including early TNM stage for tumors [1], good performance status (PS) on the Karnofsky Performance Index or the Eastern Cooperative Oncology Group (ECOG) scale [3, 4], female sex [5], young age [3, 4], and non-neuroendocrine characteristics in histology [6].

Mutations in the epidermal growth factor receptor (EGFR) kinase domain of lung adenocarcinoma have been viewed as the most reproducible predictive factor for susceptibility to first-generation EGFR tyrosine kinase inhibitors (TKIs) [7]. Afatinib is an irreversible ErbB family blocker and a second-generation EGFR-TKI, and its efficacy as first-line treatment for patients with advanced *EGFR* mutation-positive lung adenocarcinoma has subsequently been proven [8–10].

Up to 20% of patients with NSCLC present with central nervous system (CNS) metastases at the time of first diagnosis [11, 12]. The use of any first- or second-generation EGFR-TKIs alone for the treatment of brain metastases in patients with *EGFR* mutant-positive lung adenocarcinoma showed a favorable cerebral response rate of > 50% [13–16]. The efficacy of EGFR-TKIs for controlling brain metastases has been shown to be similar to that of surgical resection, stereotactic radiosurgery (SRS), or whole-brain radiotherapy (WBRT) [17, 18].

In our previous study [19], brain metastases were found to be a significant prognostic factor for progression-free survival (PFS) in patients with *EGFR* mutation-positive lung adenocarcinoma receiving afatinib as first-line treatment. Well-designed, randomized controlled trials can prove the efficacy of the study drugs in terms of response rate and PFS, but a single-center study can demonstrate the overall survival (OS) benefit of the drug in the study population. Prognostic factors can be studied in real-world patient settings. As such, we extended our previous research [19] and analyzed a real-world cohort of patients treated with first-line afatinib. Further, we investigated the prognostic factors in this specific population.

## RESULTS

### Demographics and clinical variables of the study

**Figure 1:**
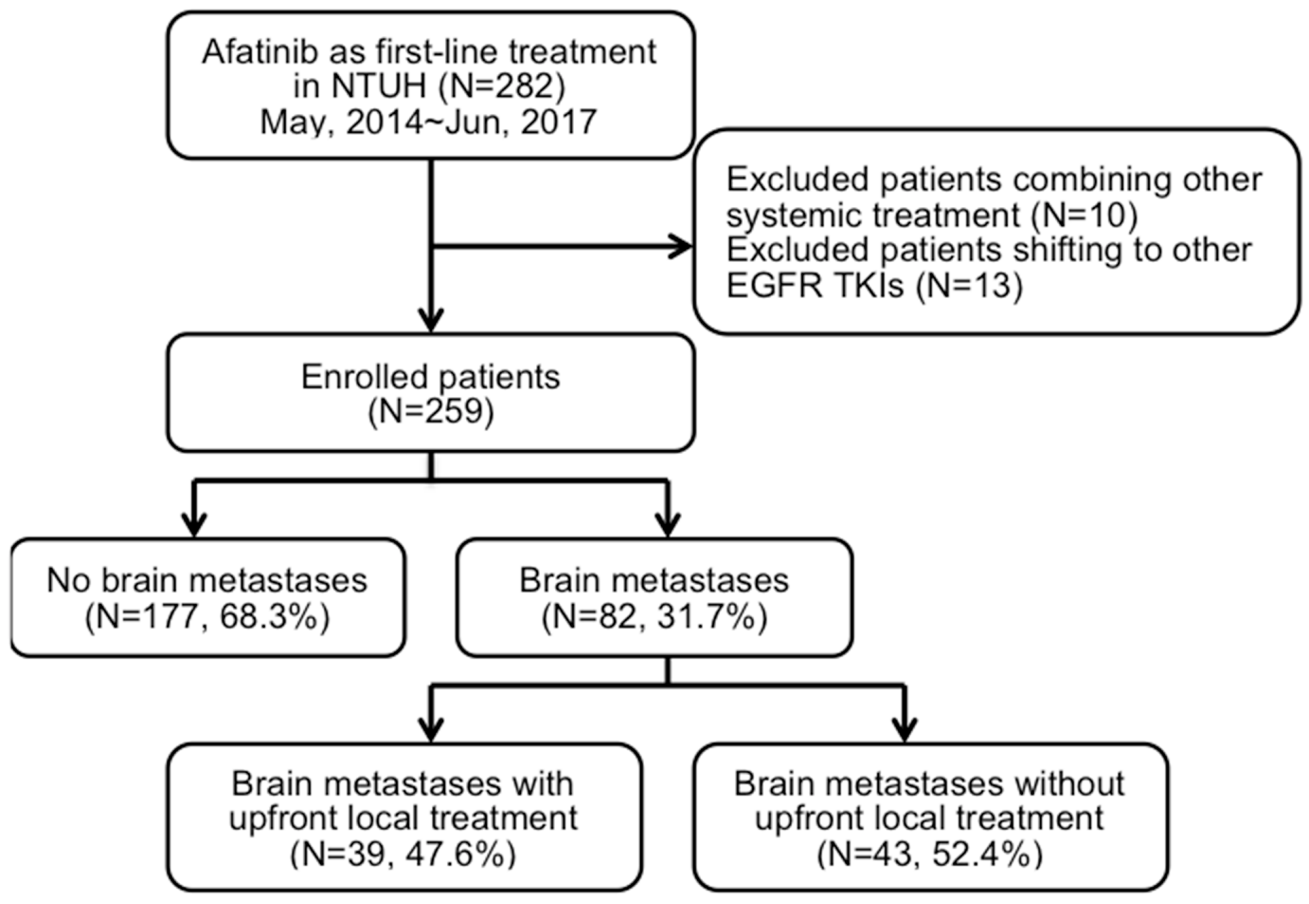
Flow chart of patient recruitment

This is an extended study from our previous cohort research, and part of the results had been published [19]. We retrospectively recruited this study cohort from the Taiwan National Health Insurance approved list of afatinib applicants from May 2014 to June 2017 at the National Taiwan University Hospital, a tertiary medical center in Taiwan. A total of 282 patients were screened, and 259 met the inclusion criteria of advanced *EGFR* mutation-positive lung adenocarcinoma with afatinib as a first-line treatment (Figure 1). The patients’ median age was 62 (range, 28–87) years. A total of 157 patients (60.6%) were women, and 187 (72.2%) were never smokers (Table 1). Most patients had relatively good PS, and only 19 patients (7.4%) had an ECOG performance score of ≥ 2. The enrolled patients were divided into three groups according to their *EGFR* mutation status as defined in the previous study: Group 1, “classical” mutation; Group 2, complex mutation with classical mutation; and Group 3, rare mutation with or without complex mutation. Majority of the patients belonged to Group 1 (n = 207, 79.9%). A total of 151 (58.3%) patients had exon 19 deletion, while 53 (20.5%) had p.L858R mutation.

**Table 1:** Clinical characteristics and comparison of patients receiving afatinib according to brain metastases

Characteristic	Patients receiving afatinib as first-line treatment			*P*-value
	All	With BM	Without BM	
	(*n* = 259)	(*n* = 82)	(*n* = 177)	
Age (years), median (range)	62 (28–87)	61 (31–82)	62 (28–87)	
Sex, *n* (%)				0.724
M	102 (39.4)	31 (37.8)	71 (40.1)	
F	157 (60.6)	51 (52.2)	106 (59.9)	
Smoking status, *n* (%)				0.846
Never smoked	187 (72.2)	61 (74.4)	126 (71.2)	
Ex-smoker^a^	26 (10.0)	8 (9.8)	18 (10.2)	
Current smoker	46 (17.8)	13 (15.9)	33 (18.6)	
Baseline ECOG PS, *n* (%)				<0.001^*^
0	77 (29.7)	21 (25.6)	56 (31.6)	
1	163 (62.9)	46 (56.1)	117 (66.1)	
2–4	19 (7.4)	15 (18.3)	4 (2.3)	
*EGFR* mutation status, *n* (%)				0.895
Group 1 (Classical mutation[s])	207 (79.9)	65 (79.3)	142 (80.2)	
19DEL	151 (58.3)	49 (59.8)	102 (57.6)	
p.L858R	53 (20.5)	14 (17.1)	39 (22.0)	
p.L858R and 19DEL	3 (1.2)	2 (2.4)	1 (0.6)	
Group 2 (Complex mutation with classical mutation)	11 (4.2)	3 (3.7)	8 (4.5)	
p.L858R and p.T790M	5 (1.9)	2 (2.4)	3 (1.7)	
Other	6 (2.3)	1 (1.3)	5 (2.8)	
Group 3 (Rare mutation with or without complex mutation)	41 (15.8)	14 (17.1)	27 (15.3)	
p.L861Q	14 (5.4)	4 (4.9)	10 (5.6)	
p.G719X	11 (4.2)	4 (4.9)	7 (4.0)	
20-INS	3 (1.2)	1 (1.2)	2 (1.1)	
p.G719A and p.T790M/Others	13 (5.0)	5 (6.1)	8 (4.5)	

^*^P < 0.05.

^a^Ceased smoking > 1 year before diagnosis.

BM, brain metastases; cStage, clinical stage; DEL, deletion; ECOG, Eastern Cooperative Oncology Group; F, female; INS, insertion; EGFR, epidermal growth factor receptor; M, male; PS, performance status; SD, standard deviation.

Approximately 181 patients (69.9%) received 40 mg afatinib as initial dose. Of them, 97 (53.7%) tolerated the 40 mg dose through the first 6 months. We also determined the treatment responses to afatinib through imaging studies and by reviewing patients’ medical records (Table 2). A total of 180 (69.5%), 60 (23.2%), and 19 (7.3%) patients had partial response, stable disease, and progressive disease, respectively.

**Table 2:** Comparison of afatinib dose and effects according to brain metastases

Variable	Patients with afatinib as first-line treatment			*P*-value
	All	With BM	Without BM	
	(*n* = 259)	(*n* = 82)	(*n* = 177)	
Initial dose with 40 mg, *n* (%)	181 (69.9)	58 (70.7)	123 (69.5)	0.84
40 mg in the first 6 months, *n* (%)	139 (53.7)	45 (54.9)	94 (53.1)	0.79
Initial tumor response to afatinib treatment, *n* (%)				0.211
PR	180 (69.5)	52 (63.4)	128 (72.3)	
SD	60 (23.2)	21 (25.6)	39 (22.0)	
PD	19 (7.3)	9 (11.0)	10 (5.6)	
**Disease progression events after treatment, *n***	**148 (57.1)**	**54 (65.9)**	**94 (53.1)**	0.054
CNS progression, *n* (%)	47 (18.1)	27 (32.9)	20 (11.3)	<0.001^*^
Non-CNS progression, *n* (%)	101 (39.0)	27 (32.9)	74 (41.8)	

BM, brain metastases; CNS, central nervous system; PD, progressive disease; PR, partial response; SD, stable disease.

**Figure 2:**
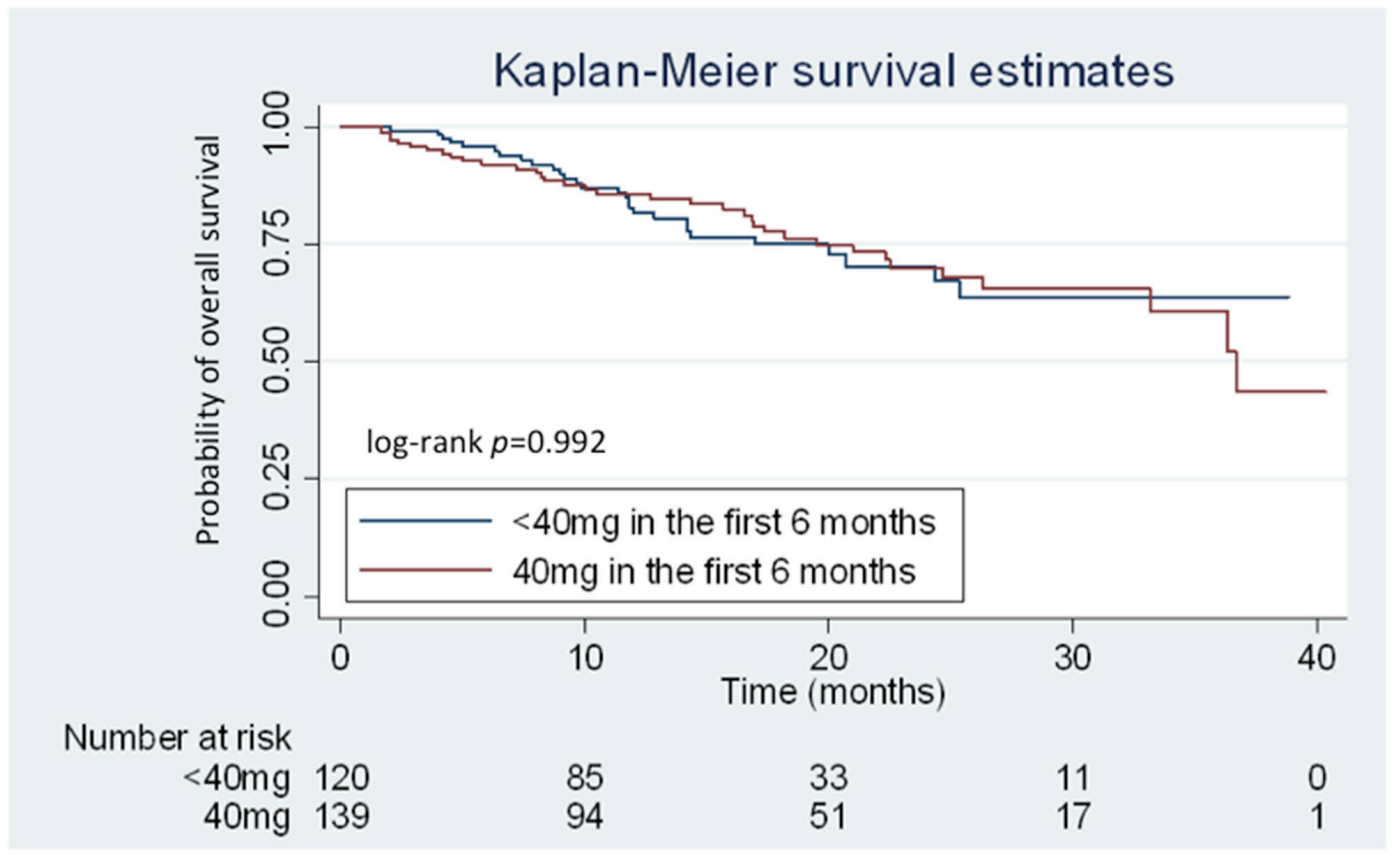
Kaplan–Meier curves for overall survival (OS) according to the treatment dose of afatinib Patients receiving 40 mg and < 40 mg of afatinib during the first 6 months are represented by the red and blue lines, respectively.

After a median follow-up duration of 22 months, the median PFS of the total patient population was 12.8 (95.0% confidence interval [CI]: 11.1–14.5) months, and the median OS was 36.7 months. Concurrently, PFS (median: 13.2 [95.0% CI: 11.2–15.1] *vs.* 12.5 [95.0% CI: 9.7–15.3] months, respectively; *P* = 0.865) and OS (median: 36.7 [95.0% CI: 32.1–41.2] months *vs.* not reached, respectively; *P* = 0.992) was not significantly different between patients who received 40 mg and < 40 mg afatinib in the first 6 months of treatment (Figure 2). However, patients with a partial response after afatinib treatment had longer OS compared to those with stable or progressive disease (both groups did not reach median duration, *P* < 0.001).

### Brain metastases as a poor prognostic factor

**Figure 3:**
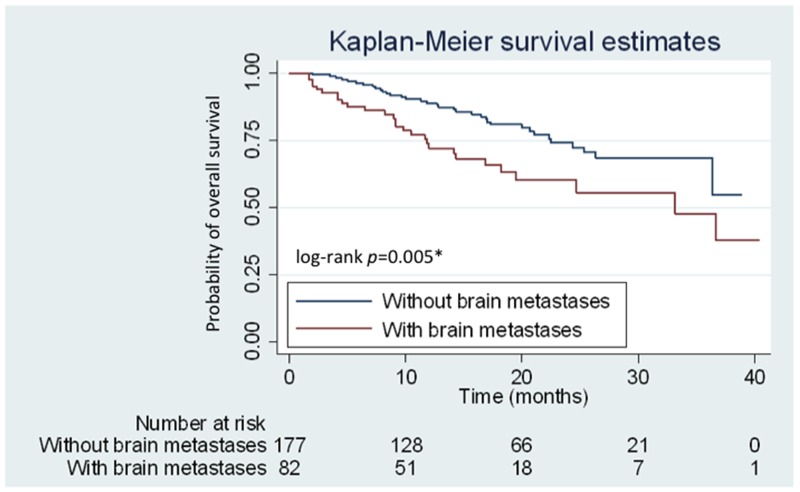
Kaplan–Meier curves for overall survival (OS) of patients with and without brain metastases Patients with and without brain metastases are represented by red and blue lines, respectively.

Of the 259 eligible patients, 82 (31.7%) were identified to have brain metastases at baseline. Patients were further subdivided into two subgroups according to the presence or absence of brain metastases at the initial clinical staging workup. No significant differences in various clinical characteristics, including age, smoking status, and *EGFR* mutation patterns, were observed between the two subgroups. Patients with brain metastases had poorer ECOG PS compared with those without brain metastases (*P* < 0.001) (Table 1). However, the initial dose preference (40 mg or < 40 mg of afatinib) and using 40 mg dosage in the entire first 6 months were not different between the two groups (*P* > 0.05) (Table 2). Meanwhile, patients with initial brain metastases had significantly higher incidence of central nervous system (CNS) progression (*P* < 0.001) (Table 2) and a shorter OS than those without brain metastases (median: 33.8 months vs. not reached, respectively; *P* = 0.005; hazard ratio [HR]: 2.03; 95.0% CI: 1.23–3.35) (Figure 3).

Those 82 patients with baseline brain metastases were subdivided into two groups according to the dose of afatinib during the first 6 months of treatment (40 mg versus <40 mg). There were no significant differences in gender, body mass index (BMI), body surface area (BSA), and ECOG PS between the two groups. Furthermore, the dose of afatinib during the first 6 months of treatment (40 mg versus <40 mg) did not make differences in clinical outcomes, including response rate (62.2% *vs.* 64.9% respectively; *P* = 0.805), PFS (median: 11.3 [95.0% CI: 8.0–14.6] *vs.* 9.0 [95.0% CI: 6.9–11.1] months, respectively; *P* = 0.766) and OS (median: 33.2 [95.0% CI: 15.6–50.7] months *vs.* not reached, respectively; *P* = 0.352) (Supplementary Table 1).

### Treatment modalities in patients with brain metastases

Eighty-two patients (31.7%) presented with brain metastases at the time of initial diagnosis (Figure 1), and 6 (7.3%) of them presented with leptomeningeal carcinomatosis. To determine the clinical factors affecting survival, we subdivided these 82 patients according to whether or not they received upfront local treatment to brain lesions (Table 3). In terms of clinical characteristics, no significant differences in age, smoking status, and *EGFR* mutation patterns were found between these two groups. Patients with brain metastases who initially presented with poor PS, neurological symptoms, > 3 cm brain tumor, or intracranial hemorrhage tended to receive upfront local treatment.

**Table 3:** Characteristics of patients with brain metastases (BMs) who did and did not receive upfront local treatment for brain lesions

Characteristic	Patients with BMs with upfront local treatment	Patients with BMs without upfront local treatment	*P*-value
	(*n* = 39)	(*n* = 43)	
Sex, *n* (%)			0.567
M	16 (41.0)	15 (34.9)	
F	23 (59.0)	28 (65.1)	
Smoking status, *n* (%)			0.521
Never smoked	27 (69.2)	34 (79.1)	
Ex-smoker^a^	4 (10.3)	4 (9.3)	
Current smoker	8 (20.5)	5 (11.6)	
ECOG PS, *n* (%)			< 0.001^*^
0	5 (12.8)	16 (37.1)	
1	21 (53.8)	25 (58.1)	
2–4	13 (33.4)	2 (4.7)	
Presence of neurological symptoms, *n* (%)	23 (59.0)	5 (11.6)	< 0.001^*^
Patterns of BMs, *n* (%)			
Single metastasis	14 (35.9)	8 (18.6)	0.078
The largest tumor > 3cm in diameter	12 (30.8)	0	< 0.001^*^
Intracranial hemorrhage	4 (10.3)	0	<0.001^*^
Leptomeningeal carcinomatosis	1 (2.6)	5 (11.6)	0.115
*EGFR* mutation status, *n* (%)			0.361
Group 1^c^	29 (74.4)	36 (83.7)	
Group 2^d^	1 (2.6)	2 (4.7)	
Group 3^e^	9 (23.1)	5 (11.6)	

^*^P < 0.05.

^a^Ceased smoking >1 year before diagnosis.

^b^Significant weight loss of >10.0% within 6 months of diagnosis.

^c^Classical mutation(s).

^d^Complex mutation with classical mutation.

^e^Rare mutation with or without complex mutation.

BM, brain metastases; ECOG, Eastern Cooperative Oncology Group; EGFR, epidermal growth factor receptor; F, female; M, male; PD, progressive disease; PS, performance status.

**Figure 4:**
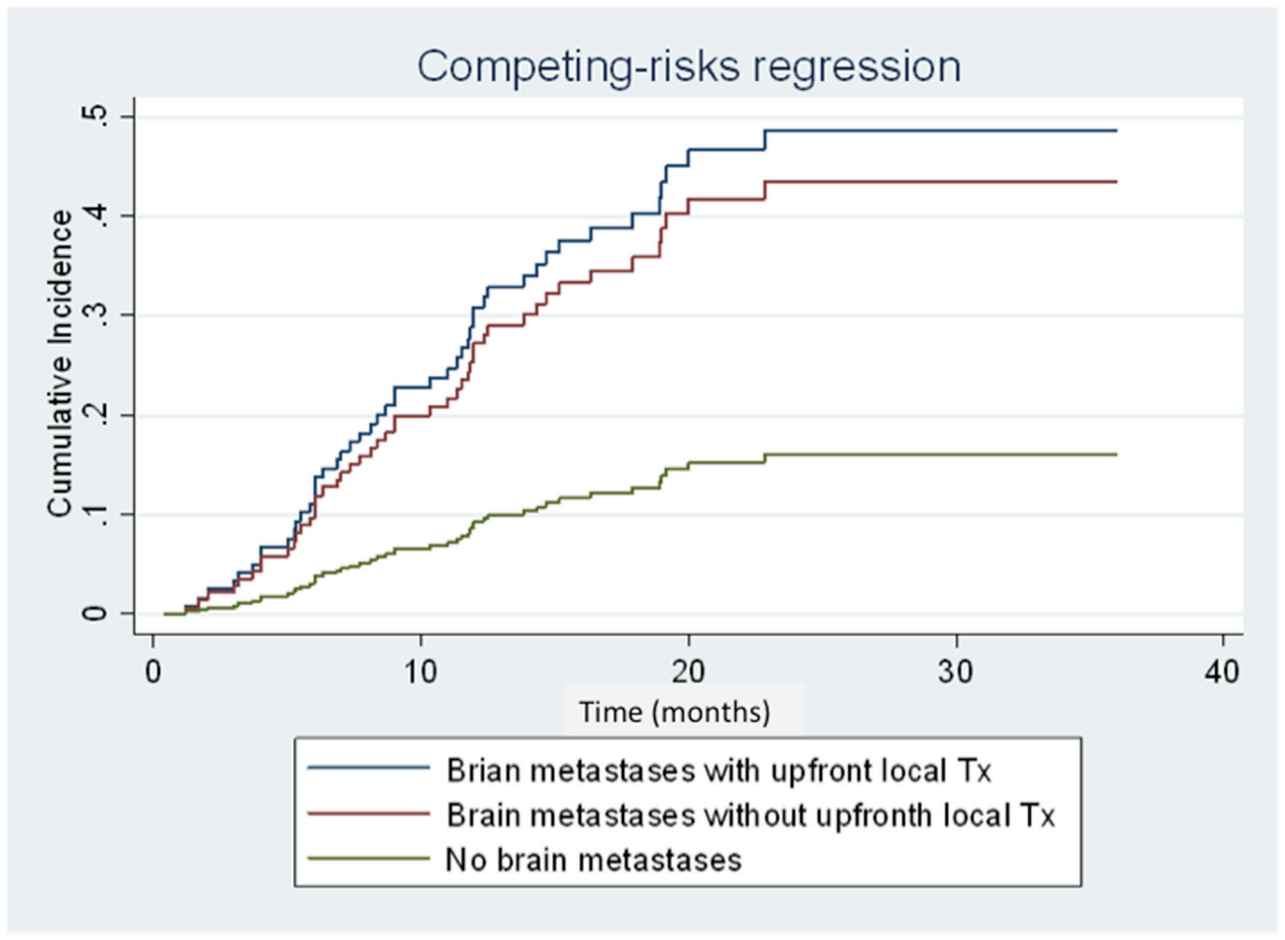
Cumulative incidence curves of central nervous system (CNS) progression of patients with brain metastases with/without upfront local treatment and patients without brain metastases at diagnosis Patients with brain metastases with/without upfront local treatment and patients without brain metastases are represented by the blue, red, and green lines, respectively.

**Figure 5:**
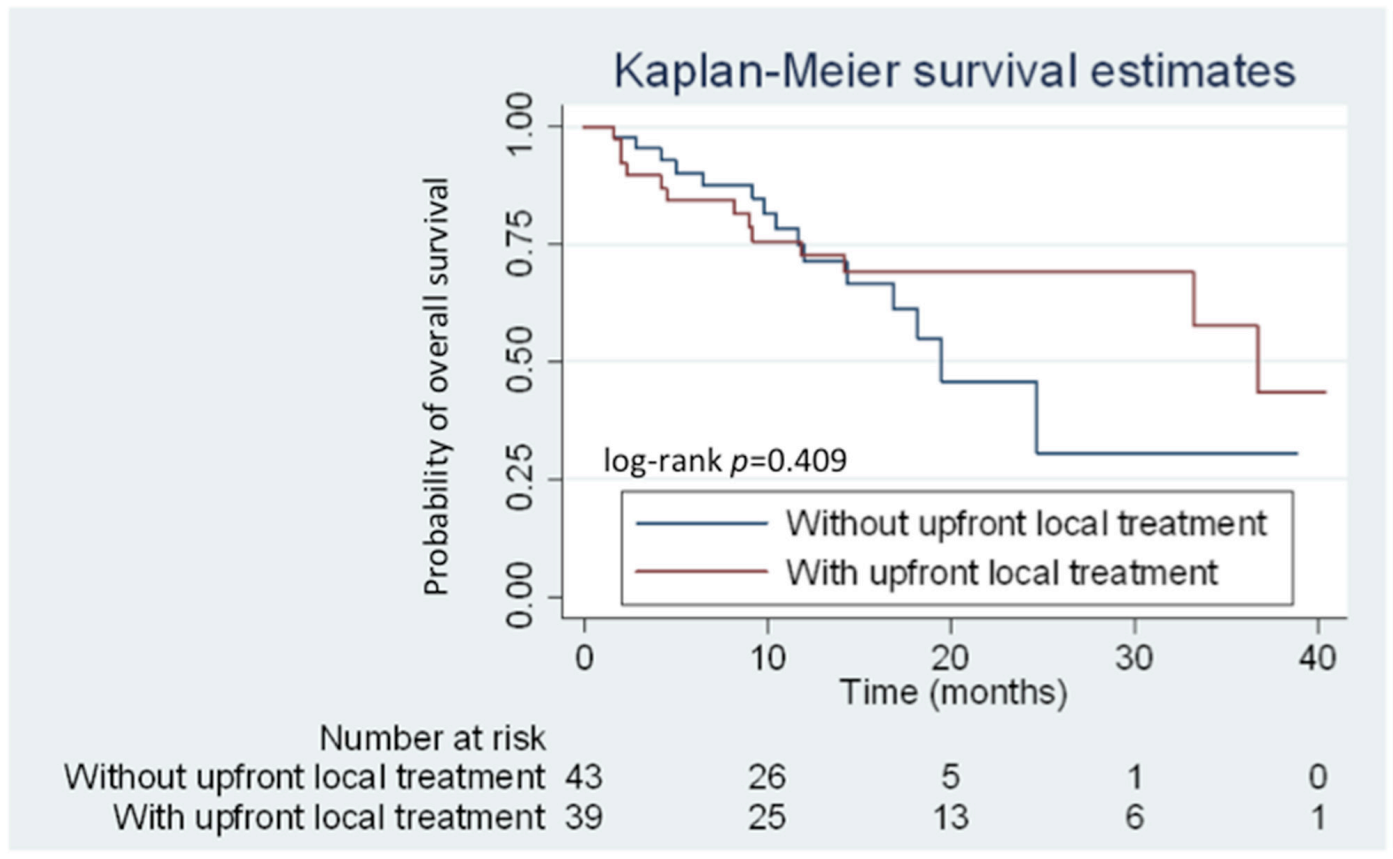
Kaplan–Meier curves for overall survival (OS) of patients with and without upfront local treatment Patients with and without upfront local treatment are represented by the red and blue lines, respectively.

Competing risk regression analysis revealed that brain metastasis at initial diagnosis was associated with high risk for CNS progression (subhazard ratio: 3.34; 95.0% CI: 1.68–6.67; *P =* 0.001) (Figure 4). However, no difference in cumulative incidence of CNS progression (subhazard ratio: 1.16; 95.0% CI: 0.54–2.50; *P =* 0.70) (Figure 4) and in OS (median 36.7 *vs.* 19.5 months, respectively; (HR: 0.73; 95.0% CI: 0.34–1.56; *P* = 0.409) (Figure 5) was noted between patients with initial brain metastases who did and did not receive upfront local treatments.

No standard treatment modalities for neurologically asymptomatic brain metastases in patients with *EGFR* mutation have been established. We analyzed those neurologically asymptomatic patients in our study (n = 54) who received any upfront local treatment (n = 16) and found that such treatment had no significant OS benefit compared with those without upfront local treatment (median OS: 33.1 *vs.* 19.5 months, respectively; *P* = 0.728) (HR: 0.84; 95.0% CI: 0.31–2.28).

### Univariate and multivariate analyses of overall survival

We used univariate and multivariate analyses to determine the influence of clinical factors on OS of afatinib-treated patients in our real-world cohort study. The results of multivariate analyses showed that clinical variables, including sex, smoking status, *EGFR* mutation status, and 40 mg or < 40 mg of afatinib dose in the first 6 months, were not associated with OS, and the most significant prognostic factors influencing OS were existence of brain metastases and treatment responses at initial evaluation (Table 4).

**Table 4:** Univariate and multivariate analyses of clinical factors for overall survival in a real-world cohort

Clinical factor	Patients (*n*)	Univariate analysis		Multivariate analysis	
		HR (95.0% CI)	*P*-value	HR (95.0% CI)	*P*-value
Sex					
M	102	1.33 (0.81–2.20)	0.26	–	–
F	157	1		–	
Smoking status					
Never smoked	187	1		–	
Current or Ex-smoker	72	1.52 (0.91–2.56)	0.112	–	–
ECOG PS					
0–1	239	1		–	
2–4	20	1.50 (0.68–3.31)	0.313	–	–
BMs					
Present	82	2.03 (1.23–3.35)	0.006^*^	1.97 (1.19–3.26)	0.008^*^
Absent	177	1		1	
*EGFR* mutation status					
Group 1^a^	207	0.58 (0.36–1.00)	0.05	0.59 (0.34–1.02)	0.06
Group 2–3^b, c^	52	1		1	
Tumor response to afatinib treatment					
PR	180	0.43 (0.26–0.71)	0.001^*^	0.47 (0.28–0.77)	0.003^*^
SD/PD	79	1		1	
Afatinib dose during the first 6 months of treatment (mg)					
40	139	0.997 (0.61–1.64)	0.992	–	–
< 40	120	1		–	

^*^P < 0.05.

^a^Classical mutation(s).

^b^Complex mutation with classical mutation.

^c^Rare mutation with or without complex mutation.

BM, brain metastasis; CI, confidence interval; ECOG, Eastern Cooperative Oncology Group; EGFR, epidermal growth factor receptor; F, female; HR, hazard ratio; M, male; PD, progressive disease; PR, partial response; PS, performance status; SD, stable disease.

## DISCUSSION

The median PFS and OS of patients with *EGFR* mutations were 12.8 and 36.7 months, respectively. The absence of brain metastases and response to afatinib treatment was associated with better OS. Clinically, ECOG PS status is considered an important prognostic factor for OS of patients with *EGFR* mutation-positive lung cancer [4]. In our study, the enrolled patients without brain metastases had a relatively good ECOG PS compared with those with brain metastases, but PS status in afatinib as first-line treatment in patients with *EGFR* mutation-positive lung adenocarcinoma did not yield a difference in OS after multivariate analyses.

In clinical practice, oncologists and neurological surgeons prescribe upfront local therapy for patients presenting with any significant neurological symptoms, intracranial hemorrhage, and those with > 3 cm brain tumor on imaging studies. Although patients who received upfront local treatment initially had relatively poor PS, combined upfront local treatment and afatinib can provide a good median OS. A multi-institutional study in the United States also demonstrated that the use of upfront SRS or WBRT is associated with better OS (46 and 30 months, respectively) in patients with *EGFR*-mutant NSCLC with brain metastases compared to EGFR-TKI monotherapy [20]. A high cerebral efficacy of EGFR-TKIs has been reported in recent studies, with high response rates of 70% to 80% in patients with *EGFR-*mutant NSCLC with CNS metastases [21–23]. However, the clinical efficacy of such drug in terms of intracranial responses can be confounded by the varying concentration of different TKIs in the cerebrospinal fluid (CSF) [24, 25]. WBRT might disrupt the blood-brain barrier, causing a favorable CNS response rate by increasing the CSF concentration in afatinib treatment. From this study, we used the analytic model of competing risk regression for investigating the cumulative incidence of CNS progression between patients receiving upfront local treatments for initial brain metastases and those did not receive upfront local treatments. Although, patients with any upfront local treatment prior to afatinib could have better OS (median 36.7 *vs.* 19.5 months, respectively) compared with those without upfront local treatments, the statistical difference was not significant (HR: 0.73; 95.0% CI: 0.34–1.56; *P* = 0.409). Meanwhile, patients with brain metastases receiving any upfront local treatment prior to afatinib did not provide benefits in CNS progression (subhazard ratio: 1.16; 95.0% CI: 0.54–2.50; *P =* 0.70). The effectiveness and survival benefits of patients with upfront local treatments to brain metastases will need more clinical studies to clarify.

In conclusion, brain metastasis is associated with poor ECOG PS, high rate of CNS progression, and short OS. Brain metastases at the time of the diagnostic stage and the initial treatment response to afatinib are two important prognostic factors for the overall survival of first-line afatinib treated *EGFR* mutation-positive lung adenocarcinoma.

## MATERIALS AND METHODS

### Patients and data collection

From May 2014 to June 2017, we subsequently retrieved data of patients with advanced *EGFR* mutation-positive lung adenocarcinoma receiving afatinib as a first-line therapy at the National Taiwan University Hospital (Taipei, Taiwan). We excluded patients (1) with unknown *EGFR* mutation status, (2) those in whom afatinib was discontinued and treatment was subsequent switched to other EGFR-TKIs prior to disease progression, and (3) those who received a combination of immunotherapy or palliative chemotherapy prior to afatinib treatment. This study had been approved by the Research Ethics Committee of the National Taiwan University Hospital (Taipei, Taiwan), and written informed consent was waived owing to the retrospective nature of the study.

Of the 282 screened patients, 23 patients were excluded because they received systemic therapy or immune checkpoint therapy concomitantly (n = 10) and afatinib was discontinued and then switched to other EGFR-TKI treatment due to various severe adverse events (n = 13); including 5 severe skin rashes, 5 oral mucositis, 1 refractory diarrhea, 1 paronychia, and 1 interstitial pneumonitis. The clinical characteristics, imaging studies, and medical records of the 259 enrolled patients were consecutively collected until August 2017. Ex-smokers were defined as patients who quit smoking for > 1 year at the time of lung cancer diagnosis. ECOG scale was used to determine the patients’ PS [26]. All patients with stage IV lung adenocarcinoma [27] received afatinib as first-line treatment.

Tumor responses to afatinib were evaluated and recorded by primary care physicians according to the Response Evaluation Criteria in Solid Tumors (version 1.1) [28]. Imaging studies, including computed tomography of the chest and computed tomography/magnetic resonance imaging of brain, were routinely followed up at a 3-month interval. PFS was defined as the period from starting afatinib treatment to the point of radiologically documented disease progression. OS was defined as the duration from commencing afatinib treatment to death from any cause.

The cutoff date for collecting clinical data ended on August 31, 2017. The median follow-up period was 22.0 (range, 2.0–40.0) months.

### Data on *EGFR* mutation status

Data on the *EGFR* mutation status of patients were collected from formal pathology reports and referral data from other hospitals. Cancer specimens could be obtained from primary lung adenocarcinomas, tissues from metastatic sites, or malignant pleural effusion cell blocks.

### Statistical analysis

In our study, categorical variables were compared using Pearson's chi-squared tests for. OS was calculated using the Kaplan—Meier method and compared using the log-rank test. We also used cumulative incidence risk to analyze the first events of CNS progression. Other first events of non-CNS progression, death or loss to follow-up beyond progression were analyzed as competing risks. HRs were used in Cox proportional hazard models, and the corresponding 95% CIs were used to compare the OS between the treatment subgroups. The Statistical Package for the Social Sciences for Windows software version 18.0 (SPSS Inc., Chicago, IL, USA) were used for all statistical analyses. OS and cumulative incidence curves were plotted using STATA for Windows software version 14.0 (StataCorp, College station, TX, USA). A two-sided *P* < 0.05 was considered statistically significant.

## SUPPLEMENTARY MATERIALS FIGURES AND TABLES



## Abbreviations

CI: confidence interval
ECOG: Eastern Cooperative Oncology Group
EGFR-TKI: epidermal growth factor receptor-tyrosine kinase inhibitor
HR: hazard ratio
OS: overall survival
PFS: progression-free survival
PS: performance status
