# Supplementary material for: Prognostic factors of afatinib as a first-line therapy for advanced EGFR mutation-positive lung adenocarcinoma: a real-world, large cohort study
Source: Oncotarget. 2018 May 4;9(34):23749–60. doi: 10.18632/oncotarget.25255 (PMC5955108; doi:10.18632/oncotarget.25255)
Supplement: Supplementary file 1 [file oncotarget-09-23749-s001.pdf]

Prognostic factors of afatinib as a first-line therapy for advanced *EGFR* mutation-positive lung adenocarcinoma: a real-world, large cohort study

SUPPLEMENTARY MATERIALS

Supplementary Table 1: Clinical characteristics and comparison of patients with BM groups according to afatinib dose

Characteristic	Patients with BM (*n* = 82)	Afatinib 40mg in first 6 months (*n* = 45)	Afatinib < 40mg in first 6 months (*n* = 37)	*P*-value
Age (years), median (range)	61 (31-82)	60 (31-82)	62 (45-81)	
Sex, n (%)				0.068
M	31 (37.8)	21 (46.7)	10 (27.0)	
F	51 (62.2)	24 (53.3)	27 (73.0)	
BMI[a], mean (SD)	23.0 (3.1)	23.0 (3.2)	23.0 (3.1)	0.917
BSA[b], mean (SD)	1.63 (0.17)	1.66 (0.19)	1.61 (0.15)	0.229
Baseline ECOG PS, n (%)				0.894
≤1	77 (81.7)	37(82.2)	30 (81.1)	
>1	15 (18.3)	8 (17.8)	7 (18.9)	
Tumor responses to afatinib, n (%)				0.805
PR	52 (63.4)	28 (62.2)	24 (64.9)	
Stable disease/PD	30 (36.6)	17 (37.8)	13 (35.1)	
Clinical outcomes				
Median PFS, months (95% CI)	9.3 (7.3-11.4)	11.3 (8.0-14.6)	9.0 (6.9-11.1)	0.766
Median OS, months (95% CI)	33.2 (19.9-46.4)	33.2 (15.6-50.7)	not reached	0.352

[*]$P < 0.05$

[a]BMI = body weight (kg)/body height (m)^2

[b]BSA= [body Height (cm) × body weight (kg)/ 3600]$^{1/2}$

BM, brain metastases; CI, confidence interval; ECOG, Eastern Cooperative Oncology Group; F, female; EGFR, epidermal growth factor receptor; M, male; OS, overall survival; PD, progressive disease; PFS, progression-free survival; PR, partial response; PS, performance status; SD, standard deviation.
